# Development of a Continuous Extrusion Process for Alginate Biopolymer Films for Sustainable Applications

**DOI:** 10.3390/polym17131818

**Published:** 2025-06-29

**Authors:** Zahra Eslami, Saïd Elkoun, Miraidin Mirzapour, Mathieu Robert

**Affiliations:** 1 Center for Innovation in Technological Ecodesign (CITE), University of Sherbrooke, Sherbrooke, QC J1K 2R1, Canada; zahra.eslami@usherbrooke.ca (Z.E.); miraidin.mirzapour@usherbrooke.ca (M.M.); mathieu.robert2@usherbrooke.ca (M.R.); 2 Research Center for High-Performance Polymer and Composite Systems (CREPEC), Montreal, QC H3A 0C3, Canada

**Keywords:** alginate, extruder, plasticizer, glass transition temperature, mechanical properties

## Abstract

This study presents a novel method for producing extrudable alginate-based films using continuous thermo-mechanical mixing, providing a scalable alternative to conventional solvent-casting techniques. The effects of glycerol concentration (30–50 wt%) and processing temperature (110–120 °C) on the films’ thermal, mechanical, and structural properties were systematically investigated. Structural characterization was performed using ^1^H NMR and FT-IR, and thermal transitions were analyzed via DSC (Differential Scanning Calorimetry) and DMA (Dynamic Mechanical Analysis). The glass transition temperature (Tg) of the alginate/glycerol/water system was modeled using the Gordon–Taylor equation. Glycerol incorporation significantly reduced Tg—by up to 76 °C with 40 wt% glycerol—and enhanced ductility and toughness, reaching 3.26 MJ/m^3^ at the optimal level. The influence of processing temperature was found to depend on plasticizer content: at lower glycerol levels, elevated temperatures decreased Tg and elongation at break, likely due to thermal degradation. However, films with higher glycerol content retained stable mechanical and thermal behavior across both temperature profiles. This work is among the first to explore how processing temperature affects extruded, plasticized pure alginate films. The findings provide key insights into the formulation and scalable production of bio-based packaging materials, highlighting the importance of optimizing both plasticizer concentration and processing parameters.

## 1. Introduction

In recent decades, polysaccharide-based polymers have garnered significant attention globally due to their renewability, biodegradability, and environmental benefits, offering an alternative to synthetic plastics [1,2,3]. Among them, alginate (a copolymer of β-D-mannuronic and α-L-guluronic acids) is widely utilized due to its excellent film-forming and gelation properties [4]. Alginate has numerous applications in diverse fields such as pharmaceuticals (e.g., wound dressings, drug delivery) [5], food processing (as a thickening and gelling agent) [6], wastewater treatment [7], and packaging [8,9].

Traditionally, alginate films are produced via solvent casting [10,11], a method suitable for laboratory-scale research but poorly suited for industrial-scale production due to limitations such as non-uniform thickness, slow drying times, and poor blend compatibility [12,13,14]. To address these challenges, this work explores an alternative, scalable approach based on thermo-mechanical extrusion, inspired by techniques commonly used in synthetic polymer processing.

Although prior studies have examined plasticized alginate and alginate-based blends processed via thermo-mechanical methods [12,14], the continuous extrusion of flexible, fully alginate-based films remains relatively underexplored. Existing literature often focuses on fixed processing conditions and lacks a systematic investigation into the combined effects of plasticizer concentration and processing temperature [15]. Moreover, the specific influence of processing temperature on the structure–property relationships of plasticized alginate films, particularly when produced via scalable extrusion techniques, has received limited attention to date.

A variety of plasticizers have been investigated to enhance flexibility and reduce the inherent brittleness of alginate-based films. Among them, sorbitol, polyethylene glycol (PEG), citrates, and glycerol have been most reported. Sorbitol, a sugar-derived alcohol, can improve mechanical strength and moisture resistance, although its propensity to crystallize over time may compromise long-term flexibility [11,16]. PEG—particularly low molecular weight grades like PEG-400—is favored for its miscibility with hydrophilic matrices; however, its application can increase the film’s sensitivity to water absorption [17,18]. Citric acid serves dual functions as both a plasticizer and a mild crosslinking agent, making its individual impact on film flexibility difficult to isolate [19,20]. Glycerol stands out among these due to its consistent plasticizing performance, strong molecular compatibility with alginate, and established safety profile, particularly in food and biomedical contexts. By enhancing chain mobility and disrupting polymer–polymer interactions, glycerol promotes flexibility without extensive structural modification of the polymer matrix [21,22]. Given its proven efficacy and widespread applicability, glycerol was selected as the plasticizer in the present study.

Glycerol is a widely used, bio-based plasticizer derived from natural sources, such as plant and animal fats, and is commonly produced as a byproduct of biodiesel manufacturing [23,24]. It functions by diffusing between polymer chains and forming hydrogen bonds with hydroxyl and carboxyl groups, thereby disrupting the native intermolecular interactions and increasing chain mobility [22,25]. This plasticizing effect reduces the glass transition temperature (Tg), decreases stiffness, and enhances the flexibility and ductility of the material. Several studies have reported improved mechanical performance of solvent-cast alginate films plasticized with glycerol, particularly in terms of elongation at break and toughness [19,26,27,28]. However, most of these works are limited to cast-film fabrication methods. The behavior of glycerol-plasticized alginate under continuous thermo-mechanical processing remains underexplored, and the combined influence of glycerol concentration and processing temperature on the structure and properties of pure alginate systems has not been systematically studied.

Processing temperature is a critical factor in the thermo-mechanical processing of polysaccharides. While moderate temperatures can improve flowability and facilitate extrusion, excessive heat may induce polymer degradation, compromising both thermal stability and mechanical performance. Unlike thermoplastics, many polysaccharides—including alginate and chitosan—degrade before reaching a molten state, which limits their processability in conventional thermal methods [29,30,31]. Moreover, they exhibit limited viscosity reduction with increasing temperature, further challenging extrusion-based fabrication. In contrast, starch-based systems undergo gelatinization in the presence of water at relatively low temperatures (typically 60–80 °C), significantly reducing viscosity and enhancing processability. For instance, potato and corn starch gelatinize between 56–66 °C and 62–72 °C, respectively [32]. Thermoplastic starch materials have been successfully produced using thermo-mechanical methods in combination with various plasticizers [33,34,35]. Some studies have explored starch/alginate blends processed via thermo-mechanical mixing [36,37,38]. However, these typically contain only small amounts of alginate (1–15 wt%), which do not substantially influence the flow behavior or viscosity of the starch matrix. In contrast, the present study focuses on developing a fully alginate-based system without incorporating other biopolymers. Achieving this requires viscosity reduction through optimized plasticization, using a combination of water and glycerol. Water is considered one of the most effective plasticizers for polysaccharides due to its ability to penetrate polymer chains and disrupt hydrogen bonding networks. Polyols such as glycerol are also effective, offering high affinity and compatibility with carbohydrate polymers [22]. However, excess plasticizer or water can lead to phase separation, stickiness, or poor mechanical performance. Therefore, identifying the optimal plasticizer concentration is essential to balance processability and final material properties [31].

Therefore, the primary objective of this study is to develop and optimize a continuously extrudable, fully alginate-based film system, using glycerol as a plasticizer and systematically varying both its concentration and processing temperature. The novelty of this work lies in the combination of continuous thermo-mechanical processing with a multi-technique characterization and modeling approach, including the use of Gordon–Taylor modeling for glass transition behavior. The outcome is intended to guide scalable production and formulation strategies for biodegradable alginate-based materials. For this purpose, the composition and M/G ratio of the biopolymer were determined using ^1^H-NMR, while the chemical structure of the plasticized alginate films was analyzed by FTIR. Thermal properties, including glass transition temperature (*T_g_*), melting behavior, and degradation temperature, were assessed using DSC, with *T_g_* also measured by DMA. Finally, the mechanical properties of the films were evaluated in detail, focusing on the effects of glycerol content and processing temperature.

## 2. Materials and Methods

### 2.1. Materials

Sodium alginate (W201502, Sigma-Aldrich, Oakville, ON, Canada) with a purity greater than 90% was used in this study. The viscosity of a 1% alginate solution in water at 25 °C ranged from 5 to 40 cps. The intrinsic viscosity of the studied alginate was 1250 cm^3^/g, measured at 25 °C, and its molecular weight was estimated to be 379,706.55 g/mol (according to our previous study) [39]. Glycerol (purity > 99%), used as a plasticizer, was supplied by Thermo Fisher Scientific (Burlington, ON, Canada). Its molecular weight is 92.09 g/mol, with a density of 1.261 g/cm^3^ and a boiling point of 290 °C. Distilled water was used for all sample preparations.

### 2.2. Film-Forming Methods

**Figure 1:**
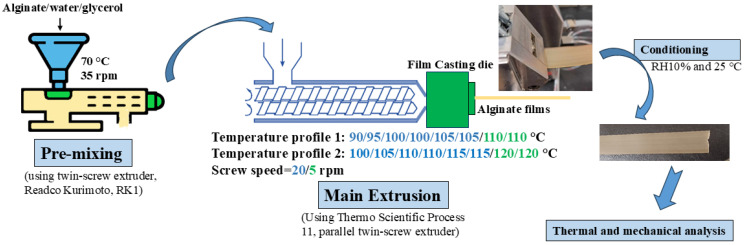
The preparation procedure for alginate-based materials using the thermo-mechanical mixing method.

The thermo-mechanical mixing method was employed to produce alginate-based films, as illustrated in Figure 1. In this method, the mixture was first processed in a twin-screw extruder RK1 (Readco Kurimoto LLC, York, PA, USA) with a mixing chamber measuring 2.54 cm × 25.4 cm. Alginate, glycerol, and water were mixed at 70 °C for 30 min at a screw speed of 35 rpm to ensure a homogeneous blend. This stage was essential to achieve a uniform mixture and prevent the delamination of the alginate-based material before feeding it into the main extruder. Additionally, the processing temperature at this stage was kept low (70 °C) to prevent material degradation. The resulting material was then fed into the main extruder (Thermo Scientific Process 11, parallel twin-screw extruder, with a barrel diameter of 11 mm and a length of 40 L/D). Two different temperature profiles were applied for processing the alginate-based films, as shown in Figure 1. A film die was mounted horizontally at the extruder exit to produce fine films with an approximate thickness of 1 mm. Although the thickness of the materials (~1 mm) exceeds the conventional definition of polymer films, the term “film” is used throughout this work due to the flexible, continuous nature of the extrudates and their relevance to film-based applications. Additionally, the screw speed for the extruder was optimized at 20 rpm, while the film die screw speed was set at 5 rpm. The resulting extrudates were homogeneous, yellowish, and transparent, with a rubber-like texture.

Pure and plasticized alginate-based mixtures with varying glycerol concentrations (0%, 30%, 40%, 45%, and 50% *w*/*w*) were produced using this method. The samples were labeled according to their glycerol weight per alginate weight (see Table 1). The amount of water incorporated was adjusted based on the glycerol content. Since glycerol is a liquid, it enhances the flowability of the mixture during extrusion, meaning that higher glycerol concentrations reduce the amount of water required to achieve a molten state. However, excessive water addition could lead to phase separation, so the water content was carefully controlled in relation to the glycerol concentration.

Films with various glycerol concentrations were produced using the first temperature profile (Figure 1), with the exit die temperature set to 110 °C. Additionally, the second temperature profile was applied to fabricate films with lower (SA-30G) and higher (SA-45G) glycerol concentrations, allowing for a comparison of the effects of temperature on the thermal and mechanical properties of the films produced under both profiles. After production and prior to characterization, the samples were conditioned at 25 °C and 10% relative humidity (RH) for four days. The H300 Series Temperature/Humidity Chamber (Folyon Technologies Inc., Toms River, NJ, USA) was used to measure the water content of the films under these conditions. After equilibration for four days, the sample weights were recorded. The difference between the weights of the moisture-equilibrated and dried samples (dried at 105 °C for 24 h) was used to calculate the moisture content of the films conditioned at 10% RH. The typical dry mass of the samples ranged from 2 to 3 g. The moisture content per dried solid (m) (*g*/*g* solid) was calculated using the following equation:

(1) $$m=\frac{m_{me}-m_{p}}{m_{p}}$$

where *m_me_* (g) is the weight of the moisture-equilibrated sample, and *m_p_* (g) is the weight of the dried sample. A balance with an accuracy of 0.0001 g was used to measure the water content, and the procedure was repeated three times for all produced samples. It is also important to note that the mass fraction of glycerol was assumed to remain constant during extrusion and after film formation, as glycerol does not volatilize under the processing conditions employed. Therefore, by subtracting the measured water content from the total mass of the film, we obtain the combined mass of alginate and glycerol. Given that the initial mass ratio of alginate to glycerol is known, we can then calculate the individual mass fractions of alginate and glycerol in the final product. The mass fractions of alginate, glycerol, and water in the films conditioned at 10% RH are shown in Table 1. This information will be used to predict the glass transition temperature of the films using the Gordon-Taylor equation.

### 2.3. Characterization Methods

#### 2.3.1. ^1^H NMR

The ^1^H NMR spectra of the sodium alginate solutions in D_2_O were obtained by Spectrometer Bruker 300 MHz Advance III (Proton Larmor frequency of 300 MHz, Bruker BioSpin Corporation, Billerica, MA, USA). For the analysis of nuclei with a resonant frequency between 19F and 109Ag at 343 K, a dual resonance probe (BBFO; 5 mm wideband HX), Bruker BioSpin Corporation, Billerica, MA, USA, with an X-channel was used. The analysis was run three times for higher accuracy.

#### 2.3.2. FT-IR

The functional groups and chemical bonds of the produced films were identified by a Perkin Elmer FT-IR spectrometer (model 4600, Boston, MA, USA). The IR spectra were collected from the frequency of 700 to 3700 cm^−1^ at a spectral resolution of 4 cm^−1^. The tests were triplicated for higher accuracy.

#### 2.3.3. DSC (Differential Scanning Calorimeter)

The thermal behavior of plasticized and unplasticized alginate-based films was investigated using a Differential Scanning Calorimeter (DSC-Q2000, TA Instruments, Inc., New Castle, DE, USA). DSC was employed to determine the polymer’s glass transition temperature (*T_g_*), melting behavior, and degradation temperature. The DSC profiles were recorded over a temperature range of −80 °C to 250 °C at a heating rate of 10 °C/min in an inert liquid nitrogen atmosphere. Each sample, weighing approximately 10 mg, was tested at least three times to ensure reproducibility.

#### 2.3.4. DMA (Dynamical Mechanical Analysis)

A Dynamic Mechanical Analyzer (DMA), Discovery DMA850 (TA Instruments, Inc.), was used to measure the glass transition temperature (*T_g_*) and investigate the viscoelastic behavior of the alginate-based films. Measurements were performed on rectangular-shaped specimens (17.5 mm × 14 mm). The oscillation amplitude was set at 20 μm, with a temperature range of −70 °C to 120 °C. Experiments were conducted using a single cantilever mode under nitrogen gas, with a heating rate of 2 °C/min and a frequency of 1 Hz. Each sample underwent three DMA tests to ensure reproducibility.

The Gordon–Taylor equation [40] (Equation (2)) was applied to the DMA data to predict the effect of glycerol on the glass transition temperature (*T_g_*) of the extruded alginate:

(2) $$Tg=\frac{x_{A}\;T_{gA}+K_{AG}x_{G}T_{gG}+K_{AW}x_{W}T_{gW}}{x_{A}+K_{AG}x_{G}+K_{AW}x_{W}}$$

where *x_A_*, *x_G_*, and *x_W_* represent the mass fractions of alginate, glycerol, and water, respectively. These values are shown in Table 1. *T_gA_*, *T_gG_*, and *T_gW_* correspond to the glass transition temperatures of pure alginate, pure glycerol, and pure water, respectively, while *K_AG_* and *K_AW_* are constants. The fitting to the Gordon–Taylor equation was performed using absolute temperatures (Kelvin). The model parameters were estimated using the method of lowest mean absolute error (*E*) (Equation (3)), assuming *T_gW_* is −135 °C [41], and T_gG_ is −87 °C [42].

(3) $$E=\frac{1}{n}\sum_{i=1}^{n}\left|T_{g,\;experimental}-T_{g,\;predicted}\right|$$

where *T_g,experimental_* is the glass transition temperature of the films obtained by DMA method, *T_g,predicted_* is the glass transition temperature calculated by Gordon-Taylor equation, and *n* is the number of the samples.

#### 2.3.5. Tensile Test

Tensile testing was performed at room temperature on dumbbell-shaped samples at a crosshead speed of 5 mm/min using a Zwick/Roell Z050 universal testing machine (ZwickRoell, Ulm, Germany), following the ASTM D-638 Type V standard [43]. The tests were conducted using a 1 kN load cell and pneumatic clamps with serrated jaw faces. The average values were recorded from five repetitions to ensure reproducibility and reliability. The Young’s modulus (GPa) was calculated from the linear section of the stress–strain curves obtained after tensile testing. The material toughness (MJ/m^3^) was determined from the integral area of the stress–strain curves.

## 3. Results

### 3.1. The Effect of Plasticizer Concentration on the Structure and Thermal and Mechanical Properties of Alginate

#### 3.1.1. Chemical Structure

**Figure 2:**
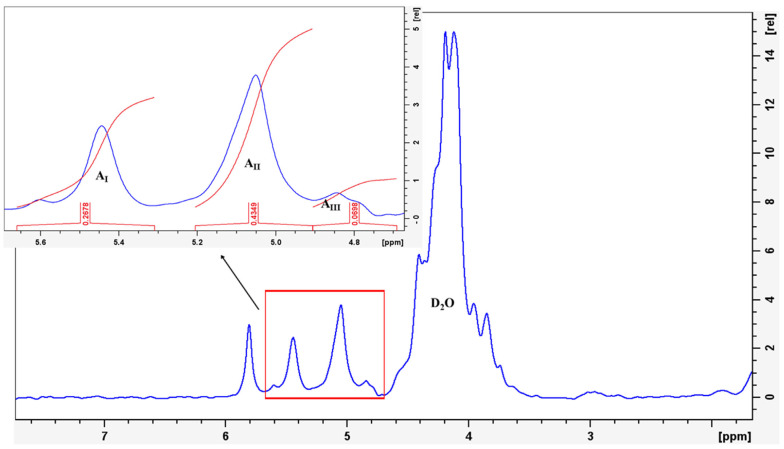
^1^H NMR spectra of the studied sodium alginate using D_2_O as a solvent.

The composition and block structure of the alginate were determined using ^1^H NMR spectroscopy (Figure 2). The molar fractions of the two monomers (G and M), as well as the four diads (GG, MM, MG, and GM), the M/G ratio (mannuronic-to-guluronic ratio), and the intrinsic viscosity (η) were calculated from the areas of three key signals (I, II, and III), as summarized in Table 2. These signals correspond to the anomeric hydrogens of G-1 (I), M-1 + GM-5 (II), and G-5 (III), which appear at 5.32–5.65 ppm, 4.92–5.20 ppm, and 4.67–4.92 ppm, respectively. In addition to the typical signals of alginic acid, an extra peak observed at 5.7–5.9 ppm, which is not characteristic of the polysaccharide structure, was identified. This peak is likely attributable to components of lower molecular weight, such as saccharose, which may have been present as an impurity in the alginate sample [10].

The equations introduced by Grasdalen et al. [44] were used to calculate F_G_, F_M_, F_GG_, F_MM_, F_MG_, and F_GM_, M/G ratio, and η value:

(4) $$F_{G}=A_{I}/(A_{II}+A_{III})$$

(5) $$F_{M}=1-F_{G}$$

(6) $$F_{GG}=A_{III}/(A_{II}+A_{III})$$

(7) $$F_{GM}=F_{MG}=F_{G}-F_{GG}$$

(8) $$F_{MM}=F_{M}-F_{MG}$$

(9) $$M/G=(1-F_{G})/F_{G}$$

(10) $$\eta =F_{MG}/(F_{M}\times F_{G})$$

The composition values presented in Table 2 show that the molar fraction of M blocks (F_M_) is lower than that of G blocks (F_G_), with the M/G ratio of alginate being less than one. This indicates that the G block predominates over the M block in the structure. Additionally, the η values exceed one, which suggests the dominance of hetero-blocks (F_MM_ and F_GG_) over homo-blocks (F_GM_ and F_MG_) in the alginate polymer [44,45,46].

**Figure 3:**
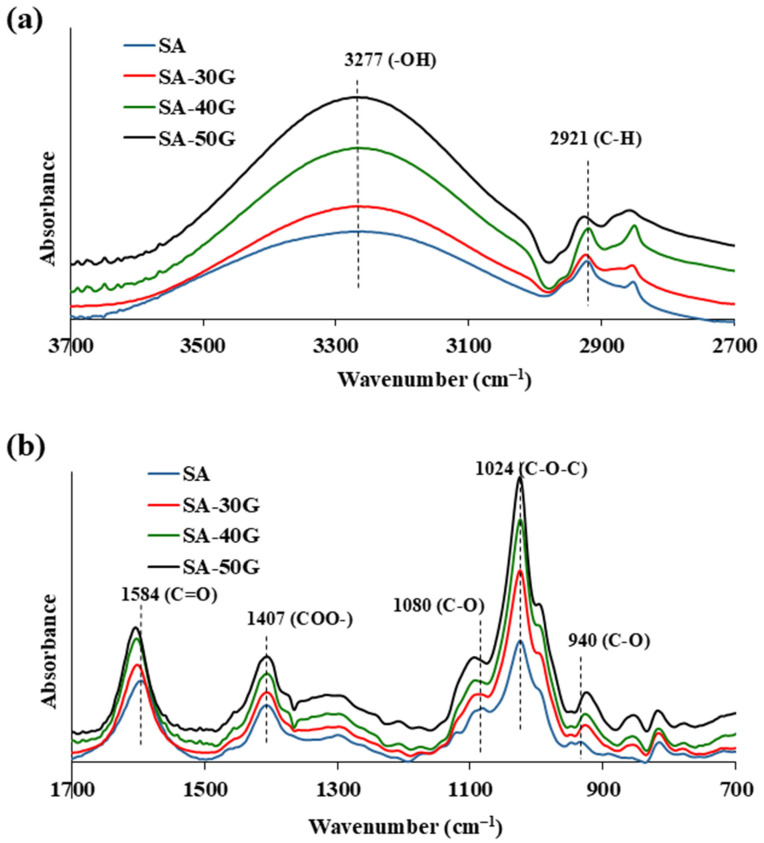
FT-IR spectrum of alginate films with different glycerol concentrations. (**a**) spectral region 2700–3700 cm^−1^ and (**b**) spectral region 700–1700 cm^−1^.

FT-IR spectroscopy was employed to investigate the interactions between glycerol and alginate, as well as to identify the key functional groups present in the films. Figure 3 displays the FT-IR spectra of alginate films with varying glycerol concentrations.

The spectrum of pure alginate exhibits several characteristic absorption bands. A broad O–H stretching vibration between 3700 and 3000 cm^−1^ corresponds to hydroxyl groups involved in hydrogen bonding. The symmetric and asymmetric C–H stretching vibrations of the CH_2_ groups appear at 2921 cm^−1^ and 2880 cm^−1^, respectively (Figure 3a). Strong absorption bands at 1584 cm^−1^ and 1407 cm^−1^ are attributed to carboxylate functional groups: the 1584 cm^−1^ band corresponds to the asymmetric stretching of the O–C–O group and C–O asymmetric vibrations of uronic acids, while the 1407 cm^−1^ peak is associated with C–OH deformation and symmetric O–C–O stretching [47,48,49]. A smaller peak at 1080 cm^−1^ corresponds to C–O stretching in glycosidic linkages of oligosaccharides. The intense peak at 1024 cm^−1^ arises from C–O and C–C stretching in the saccharide structure, particularly the guluronic acids, and includes contributions from cyclic ether (C–O–C) vibrations [50,51]. Additional bands at 941 cm^−1^, 886 cm^−1^, and 816 cm^−1^ are attributed to vibrations of α-L-guluronic and β-mannuronic acid units [47,48,52,53] (Figure 3b).

The FT-IR spectra of the plasticized alginate films retained the same general pattern as the neat alginate, but several key changes were observed with increasing glycerol content, indicating alterations in the hydrogen-bonding network. Mechanistically, glycerol molecules interact with alginate chains primarily through hydrogen bonding between their hydroxyl (–OH) groups and the carboxyl and hydroxyl groups present on the alginate backbone. The observed intensification of the broad O–H stretching band (3277 cm^−1^) with increased glycerol concentration reflects a higher population of free –OH groups from glycerol [16,52]. At the same time, the shift in the carboxylate band (1584 cm^−1^) to higher wavenumbers suggests a weakening of the original intermolecular hydrogen bonds between alginate chains, likely due to glycerol displacing water molecules and forming new, weaker hydrogen bonds with alginate. In this context, glycerol disrupts the original alginate–alginate hydrogen-bond network by inserting itself between chains, effectively increasing the interchain distance and reducing chain packing density. This disruption is further supported by the shift in the C–O stretching band (~1080 cm^−1^), which corresponds to vibrations in the glycosidic linkages, indicating a loosening of the polysaccharide network. Thus, glycerol acts not only as a physical spacer but also alters the microstructure at the molecular level, [12,16], contributing to enhanced chain mobility and reduced crystallinity observed in the thermal analyses, discussed in the following sections.

#### 3.1.2. Thermal Properties

**Figure 4:**
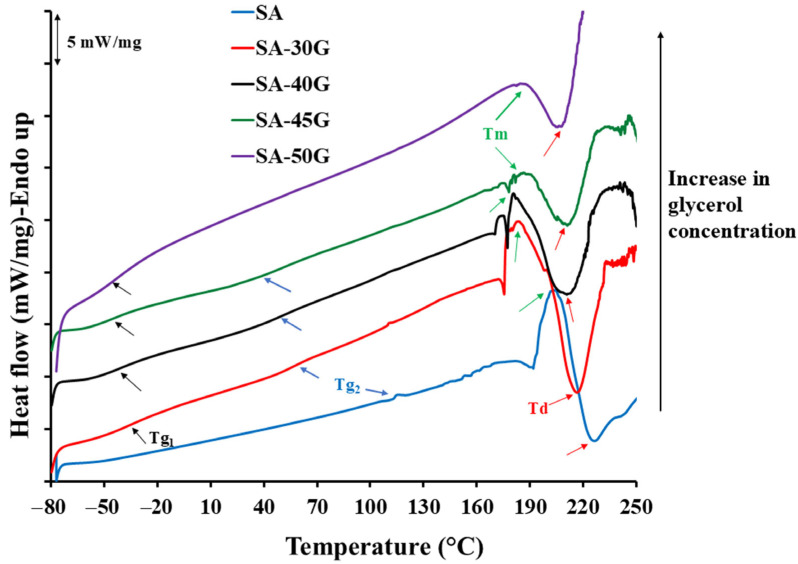
DSC scans of pure and glycerol-plasticized alginate materials.

**Figure 5:**
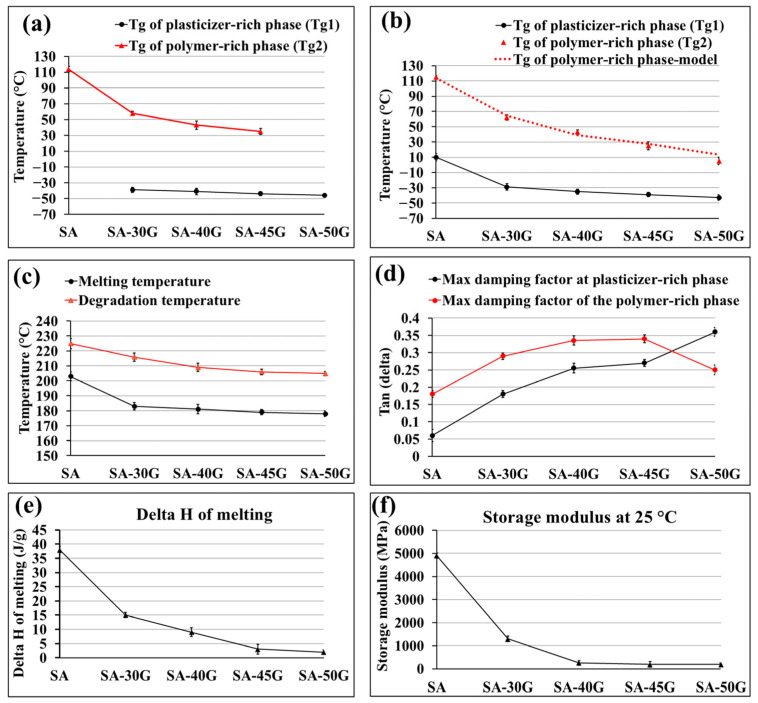
The thermal characteristics of the alginate-based materials obtained from (**a**,**c**,**e**) DSC, and (**b**,**d**,**f**) DMA results.

Figure 4 presents the DSC analysis results for pure and glycerol-plasticized alginate films produced using the first temperature profile (exit die temperature: 110 °C). The DSC curves provide insights into the glass transition temperature (*T_g_*) of the two phases, melting enthalpy and temperature, and degradation temperature. The effect of glycerol on key parameters extracted from DSC results is plotted in Figure 5a,c,e.

The DSC results indicate that the glass transition temperature of pure alginate is relatively high, at 114 °C. A small transition at lower temperatures was also observed, likely associated with low molecular weight components, impurities such as sucrose (confirmed by ^1^H-NMR analysis), as well as side chains and lateral groups of alginate. The incorporation of glycerol significantly reduced the glass transition temperature (*T_g2_*) of the polymer-rich phase, decreasing from 114 °C to 58 °C, 47 °C, and 39 °C for samples plasticized with 30%, 40%, and 45% glycerol, respectively. Additionally, in the plasticized samples, another *T_g_* at lower temperatures (*T_g_*_1_) was observed, attributed to the plasticizer-rich phase. For the sample plasticized with 50% glycerol, only a single *T_g_* at −46 °C was observed, likely due to the high plasticizing efficiency of glycerol at this concentration. The glass transition temperature of thermoplastic starch (TPS) follows the same trend as our results. Several studies have reported that TPS consists of two phases: one rich in plasticizer and the other rich in starch [37,54,55]. They concluded that when heating TPS, two *T_g_*s are expected to appear in DSC scans. The lower glass transition temperature (*T_g_*_1_) is associated with the plasticizer-rich phase, while *T_g_*_2_ is attributed to the starch-rich phase and appears at higher temperatures.

The melting temperature of alginate decreased from slightly above 200 °C for pure alginate to approximately 180 °C for plasticized samples (Figure 4 and Figure 5c). Achieving a molten state during extrusion is crucial, and plasticizers aid in reducing both the melting temperature and the viscosity of the mixture. However, the processing temperature in the extruder is maintained below the melting temperature determined by DSC. This is because the residence time inside the extruder is relatively long, and the material would degrade if processed at a higher temperature. Additionally, shear force and high pressure during processing improve the flowability of the material, allowing it to be processed at lower temperatures.

As shown in Figure 4 and Figure 5e, the melting enthalpy (ΔH) decreased significantly with increasing glycerol content. A higher ΔH of fusion indicates greater crystallinity in the material. Pure alginate, with its relatively higher crystallinity, exhibited a higher ΔH compared to the plasticized films. Glycerol disrupts the crystalline regions of alginate by penetrating the helical structure of the polysaccharide, increasing the free volume between alginate chains, and promoting the formation of an amorphous material.

The DSC analysis also revealed that the strength of polymer-polymer interactions decreased with higher glycerol concentrations. This was evident from the consistently lower peak temperatures for the exothermic degradation peaks in the plasticized films (Figure 4 and Figure 5c). A reduction in degradation temperature with the incorporation of plasticizers has been observed in other studies [10,17]. Specifically, the degradation temperature of the films plasticized with 30%, 40%, 45%, and 50% glycerol decreased by 9 °C, 16 °C, 19 °C, and 17 °C, respectively, compared to neat alginate.

**Figure 6:**
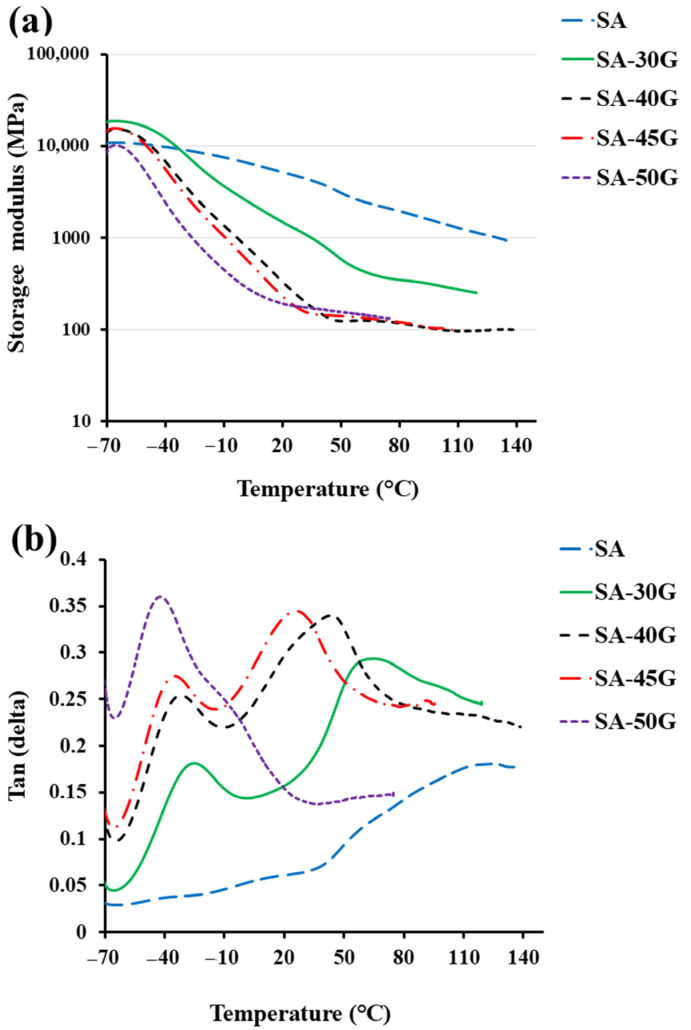
The DMA results of pure and plasticized alginate materials. (**a**) storage modulus, (**b**) Tan (delta).

DMA analysis was conducted to evaluate the effect of glycerol concentration on the viscoelastic behavior and glass transition temperatures (Tg) of alginate-based films. Figure 6 presents the storage modulus (E’) and tan δ curves for neat and plasticized alginate films. The main characteristics of the DMA are represented in Figure 5b,d,f. The results support the role of glycerol as an effective plasticizer, with clear evidence of phase-specific relaxation behavior in the composite system.

As shown in Figure 6a, neat alginate exhibited a high storage modulus (4898.2 MPa at 25 °C), with gradual softening across the tested temperature range—consistent with its rigid and hydrogen-bonded network [56,57]. In contrast, glycerol-plasticized films showed a substantial reduction in modulus and a more pronounced glass transition between −40 °C and 50 °C, indicating a transition from glassy to rubbery states. At 50% glycerol, *E’* dropped to approximately 183 MPa at 25 °C (as summarized in Figure 5f), highlighting a significant increase in flexibility. This behavior is attributed to glycerol disrupting intermolecular interactions and increasing chain mobility through hydrogen bonding and enhanced free volume [16,22].

Figure 6b shows the tan δ curves. Neat alginate displayed multiple peaks, −40 °C (attributed to low-molecular-weight components), ~10 °C (side-chain motion), ~60 °C (M-blocks), and ~115 °C (G-blocks). With glycerol incorporation, plasticized films exhibited two distinct *T_g_* peaks: *T_g_*_1_ (plasticizer-rich phase) at lower temperatures, and *T_g_*_2_ (polymer-rich phase) at higher temperatures. These reflect dual-phase relaxation behavior. For instance, in SA-30G, *T_g_*_1_ appeared at −29 °C (35 °C lower than neat alginate), while *T_g_*_2_ was observed at 59 °C, 56 °C lower than that of the neat film. With increasing glycerol content (40–50%), *T_g_*_1_ further shifted to −35 °C, −39 °C, and −43 °C, respectively. This shift corresponds to increased segmental mobility due to transient hydrogen bonding between glycerol and alginate. *T_g_*_2_ values also decreased (e.g., 46 °C at 40% and 28 °C at 45% glycerol) (as summarized in Figure 5a), indicating enhanced flexibility even in the polymer-rich domains. This behavior has been similarly reported in previous studies on plasticized alginate systems [10,58]. At 50% glycerol, however, *T_g_*_2_ disappeared entirely, replaced by a broad shoulder near −5 °C, suggesting a more homogeneous polymer–plasticizer phase and complete suppression of the rigid alginate network.

The tan δ values (damping factor) increased with glycerol content, reflecting improved energy dissipation and viscoelasticity. For neat alginate, tan δ values were 0.06 and 0.18 at *T_g_*_1_ and *T_g_*_2_, respectively. These increased to 0.18 and 0.29 in SA-30G, and up to 0.36 at 50% glycerol (Figure 5d and Figure 6), indicating greater molecular mobility and reduced network stiffness.

These DMA results confirm that glycerol effectively lowers the energy barrier for segmental motion and modifies the micro-phase morphology of alginate films. The system evolves from a rigid, hydrogen-bonded network into a more flexible, homogeneous structure with dual or merged relaxation behavior depending on the glycerol content. These changes are consistent with the observed thermal and mechanical trends and reinforce the plasticizing efficiency of glycerol in alginate matrices.

The glass transition temperature of the films at different glycerol concentrations was predicted using the Gordon–Taylor (GT) equation (Equation (2)). Figure 5b presents the experimental *T_g_* data obtained from DMA measurements, alongside the fitted GT equation for the polymer-rich phase. The results indicate that the GT equation accurately models the plasticizing effect of glycerol on the samples. The GT equation is widely used to predict the glass transition temperature of multi-component food systems and other hydrophilic biopolymers [41,59].

The model parameters (*K_AG_*, *K_AW_*, *T_gA_*—the glass transition temperature of pure alginate—and the mean absolute error (*E*)) are presented in Table 3. The *K* value reflects the diluent’s plasticizing ability, with a higher *K* value indicating better polymer plasticization. In our three-component system, the *K_AW_* value (representing alginate-water interactions) is higher than *K_AG_* (which represents alginate-glycerol interactions). This outcome was expected since water molecules (*M_w_* = 18 g/mol) are smaller than glycerol molecules (*M_w_* = 92 g/mol), allowing water to diffuse more easily between macromolecules and effectively plasticize the polymer. Additionally, the *K_AG_* value is slightly below 1, suggesting a minor heterogeneity in the alginate/glycerol system, whereas *K_AW_* is greater than 1, indicating the homogeneity of the alginate/water system [60].

On the other hand, the experimental measurement of the glass transition temperature (*T_g_*) of pure alginate is relatively difficult due to the high brittleness of alginate and the potential overlap of *T_g_* with the degradation of alginate [16,57]. However, we predicted this value to be 156 °C based on the experimental *T_g_* data and the GT model. This prediction aligns with the value reported by Russo et al. [57] for pure alginate. They observed a shoulder around 150 °C in their DMA results of completely dried alginate, which corresponds to the *T_g_* of the polymer.

#### 3.1.3. Mechanical Properties

**Figure 7:**
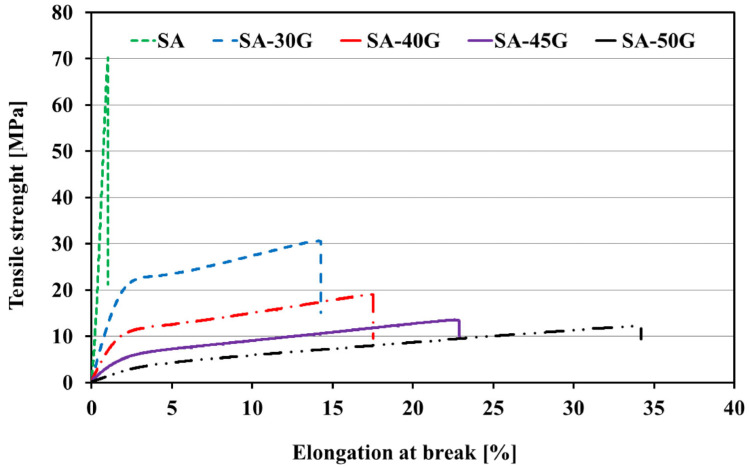
Stress–strain curves of the alginate films plasticized with different concentrations of glycerol (the films were extruded under the first temperature profile with the die exit of 110 °C).

Figure 7 presents the typical stress–strain tensile curves for pure and glycerol-plasticized alginate films extruded at 110 °C. The neat alginate film exhibited brittle fracture behavior, typical of materials with low ductility. In contrast, all plasticized films displayed stress–strain curves characteristic of ductile polymers, showing significant plastic deformation before rupture.

**Figure 8:**
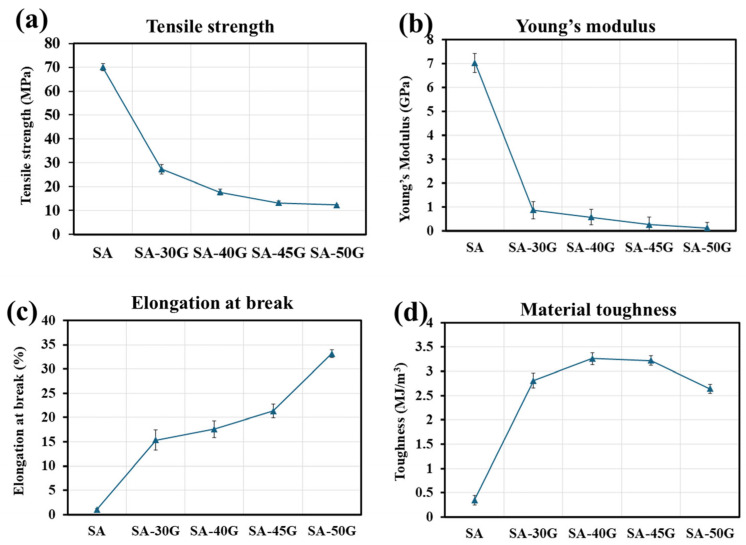
The mechanical properties of the alginate films plasticized at different concentrations of glycerol (these results are the average of at least five tests). (**a**) Tensile strength, (**b**) Young’s modulus, (**c**) Elongation at break, and (**d**) Material toughness.

The key mechanical properties, including Young’s modulus (YM), tensile strength (TS), elongation at break (EB), and material toughness were calculated from the tensile test results of the plasticized films extruded using the first temperature profile (110 °C). These values are plotted in Figure 8. Although pure alginate exhibited high TS (70 MPa) and YM (7.04 GPa), its EB was very low (1.68%), indicating brittleness. Generally, pure alginate is a very strong material with high Young’s Modulus (YM) and tensile strength (TS) [8,15,61]. For example, sodium alginate has been reported to enhance the mechanical properties of polyethylene oxide (PEO) by increasing the YM and TS of sodium alginate/PEO films produced using the solvent-casting method [61]. It was suggested that this improvement could be due to the strong hydrogen bonding between the alginate macromolecules. Additionally, pure alginate possesses a higher TS compared to commonly used thermo-processable bio-based plastics such as PLA (TS < 50–70 MPa) [62] and thermoplastic starch (TPS) (TS < 30 MPa) [63]. This makes alginate a promising alternative to conventional plastics for producing materials with comparable mechanical properties. However, the elongation at break (EB) of pure alginate is very low, though it can be increased with the incorporation of glycerol. The incorporation of glycerol significantly altered alginate properties, particularly EB. For the film plasticized with 30% glycerol, TS and YM decreased by 62% and 87%, respectively, while EB increased by approximately 800% compared to pure alginate. As the glycerol concentration increased beyond 30%, both TS and YM continued to decrease, while EB values kept rising. For the film plasticized with 50% glycerol, TS and YM dropped by 55% and 86%, respectively, while EB increased by 115% compared to the SA-30G film (Figure 8a–c). This trend is consistent with the role of glycerol as a plasticizer, which reduces hydrogen bonding and increases the free volume between alginate chains, thus enhancing the material’s flexibility and elongation. These trends align with similar studies on plasticized alginate films. Jost et al. [11] studied the effect of 20–40 wt.% glycerol on the mechanical properties of alginate films produced by the solvent-casting method. Their findings showed a similar trend, with TS decreasing and EB increasing as glycerol content rose. Similarly, Gao et al. [12], used the thermomechanical mixing method to incorporate 10–50 wt.% glycerol into sodium alginate films. They reported a significant reduction in TS and YM with increasing glycerol content, consistent with the present study. Interestingly, Gao et al. observed that EB increased with glycerol concentrations up to 30 wt.% but then decreased with higher glycerol amounts. This decrease in EB for films with high glycerol content was attributed to phase separation or segregation, where excessive glycerol disrupts the intermolecular and intramolecular bonds of the alginate, leading to premature fracture. In the present study, phase separation was not observed in the higher glycerol films, possibly because they were conditioned at a much lower relative humidity (RH 10%) compared to the 57% RH used by Gao et al. It has been shown that glycerol increases the hydrophilicity of alginate through enhanced OH-bonding [12,18], which, under higher humidity conditions, can promote phase separation. Furthermore, it is important to note that other factors, such as the source of alginate, extraction method, M/G ratio, molecular weight, film preparation method, and film thickness, can influence the mechanical properties of alginate films, as observed in different studies.

Material toughness can be defined as the energy that a material can absorb until reaching the rupture point, and it can be measured from the area under the stress–strain curve obtained from a tensile test [64]. Toughness is an important factor in the study of the mechanical properties of biological materials, as changes in toughness are the result of combined changes in tensile strength, elongation at break, and Young’s modulus. Tough materials not only exhibit considerable plastic deformation but also show significant strength. Synthetic polymer-based films typically have high toughness. For instance, the toughness of virgin low-density polyethylene (LDPE) can range from 21 ± 10 MJ/m^3^ [65]. Biopolymers, on the other hand, usually possess low toughness and can break easily under mechanical stress. Therefore, it is crucial to tailor the mechanical properties of biopolymers by incorporating plasticizers and reinforcing agents to make them comparable with the mechanical properties of synthetic counterparts.

As shown in Figure 8d, the toughness of unplasticized alginate is very low (0.35 MJ/m^3^). The toughness of pure alginate films produced by the solvent-casting method, as reported by Ma et al. [66] was 0.38 MJ/m^3^, which is very close to the value calculated in this study. With the incorporation of 30% glycerol, the toughness increased significantly by about 7 times, reaching 2.81 MJ/m^3^. Increasing the glycerol concentration to 40% resulted in an even greater increase in toughness (3.26 MJ/m^3^). The increase in the toughness of alginate-based films produced by the solvent-casting method with the incorporation of glycerol has also been reported in the research conducted by Chen et al. [67]. However, the toughness decreased slightly at higher concentrations, with samples SA-45G and SA-50G showing values of approximately 3.22 and 2.64 MJ/m^3^, respectively.

Therefore, based on the obtained results, while the sample with the highest glycerol concentration (SA-50G) offers the greatest flexibility (highest EB), the toughness of the sample with 40% glycerol is the highest among the plasticized films, indicating optimized toughness. This makes the film suitable for applications where both strength and flexibility are important.

### 3.2. The Effect of Processing Temperature on Thermal and Mechanical Properties of Alginate

The investigation of the processing temperature of biopolymers is highly important, as the optimum processing temperature is typically close to the onset of degradation temperature [13]. Although the onset of degradation temperature for plasticized alginate-based films is approximately 190–200 °C (as indicated by DSC results in Figure 4 and Figure 5), degradation in the extruder can begin at relatively lower temperatures due to higher pressure, increased shear rate, and longer residence time. It is essential to process alginate-based materials without causing decomposition to prevent the breaking of polymer chains, which would reduce the mechanical properties of the final product. However, if the processing temperature is lower than the optimum, the films may delaminate and fail to reach a molten state. Therefore, finding an optimal processing temperature for alginate-based mixtures is crucial. Rech et al. [15] reported that adjusting the extrusion temperature is critical for both the appearance and properties of the extrudate. They observed that at temperatures below 80 °C, the extrudate became sticky, opaque, and exhibited a sharkskin surface. In contrast, at temperatures of 120 °C and higher, they observed the onset of degradation. In addition, due to water evaporation the pressure inside the extruder increased. Consequently, they chose a processing temperature of 100 °C for their extrusion process.

In the present study, alginate plasticized with lower amounts of glycerol (SA-30G) and higher amounts of glycerol (SA-45G) were processed at two temperature profiles (110 and 120 °C). The torque rating during the processing of SA-30G at a die temperature of 110 °C was around 32–36%. Initially, it decreased slightly with increasing processing temperature, but it increased after 5 min and stabilized at 48–56%. This increased torque rating can be attributed to water evaporation and the increase in viscosity of the mixture as processing time increases. For the SA-45G sample, however, the increase in processing temperature led to a decrease in torque, from 24–26% to around 20–23%. This is likely due to better interaction between glycerol and the polymer at higher temperatures, which reduces the viscosity of the mixture. Processing temperature plays a key role in starch extrusion, as it needs to be high enough to gelatinize the starch and reduce the viscosity of the starch melt. Li et al. [68] investigated the effect of processing temperature (115, 135, 145, 160, and 180 °C) in a twin-screw extruder on the processability of four corn starches with different amylose contents (4.3–77.4%). They showed that, generally, torque decreased with increasing extrusion temperature. However, the variation in torque differed depending on the amylose content of the starches. For high-amylose starches, it was suggested that shear stress had a greater effect on disrupting starch granules at temperatures below 135 °C. Further increases in extrusion temperature above 145 °C did not significantly decrease torque, because the long, linear amylose chains tended to entangle, thus contributing to higher viscosity.

In the present study, the thermal and mechanical properties of the products obtained from the extrusion process were further analyzed by DSC, DMA, and tensile testing.

#### 3.2.1. Thermal Properties

**Figure 9:**
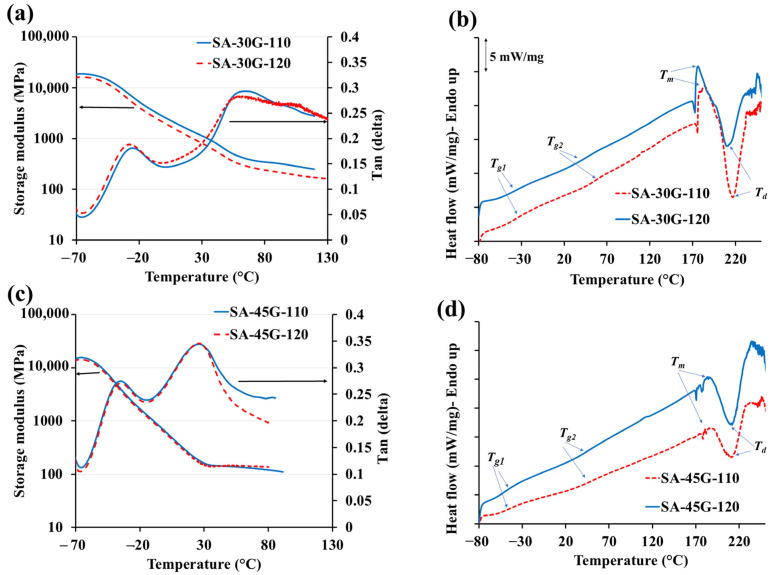
DMA and DSC scans of the films plasticized with (**a**,**b**) 30% glycerol and (**c**,**d**) 45% glycerol processed at two temperature profiles (110 and 120 °C), respectively.

Figure 9b,d compare the DSC scans of films plasticized with 30% (SA-30G) and 45% glycerol (SA-45G), processed at exit die temperatures of 110 °C and 120 °C. For SA-30G, an increase in processing temperature led to a noticeable decrease in the glass transition temperature (Tg2) of the polymer-rich phase, as well as reductions in the melting and degradation temperatures. These changes are indicative of partial thermal degradation, likely exacerbated by increased water evaporation and prolonged residence time inside the extruder.

**Figure 10:**
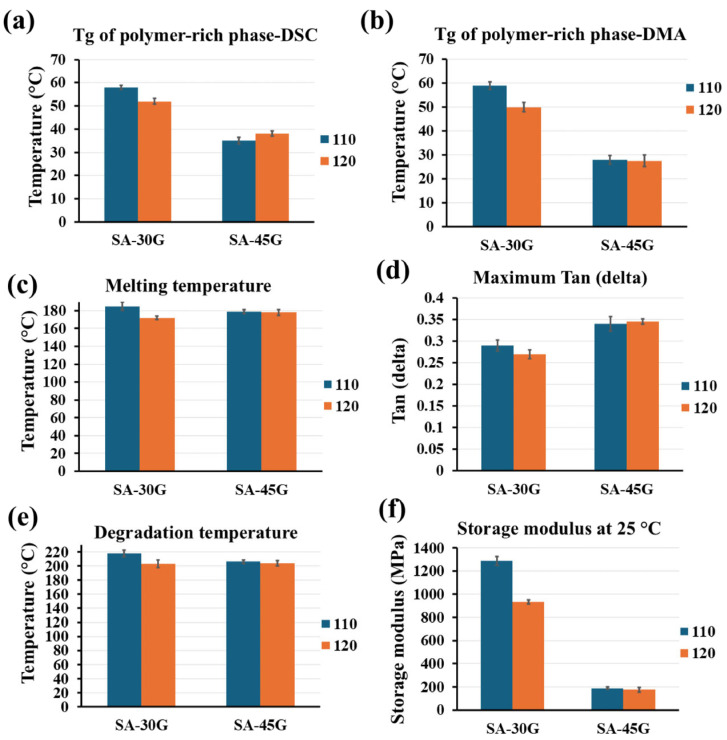
Thermal parameters of the films plasticized with 30 and 45% glycerol at two temperature profiles (110 and 120 °C) extracted from (**a**,**c**,**e**) DSC thermograms, and (**b**,**d**,**f**) DMA thermograms.

In contrast, SA-45G showed no significant change in Tg2, melting temperature, or degradation temperature across the two temperature profiles (Figure 10a,c,e), suggesting greater thermal stability at higher plasticizer content. This difference is attributed to the plasticizing dominance of glycerol, which, unlike water, is non-volatile and remains well-distributed in the matrix during processing.

Mechanistically, in low-glycerol systems, water acts as a critical co-plasticizer. At elevated temperatures, its rapid evaporation not only diminishes plasticization efficiency but also increases viscosity, leading to localized overheating and chain scission. The resulting reduction in molecular weight lowers both Tg and melting point, as confirmed by DSC thermograms. Additionally, the decrease in degradation temperature reflects the material’s heightened sensitivity to thermal stress when water content is high, and glycerol content is insufficient to buffer the effect.

In contrast, higher glycerol content mitigates these effects by maintaining molecular mobility and reducing viscosity build-up, thus preventing degradation. The stabilizing role of glycerol is further supported by its capacity to reduce shear sensitivity and distribute thermal energy more uniformly across the matrix.

DMA results (Figure 9a,c) further corroborate these findings. For SA-30G, films processed at 120 °C exhibited lower storage modulus and a reduced Tg2 compared to those processed at 110 °C (Figure 10b,d), consistent with a partially degraded, less elastic structure. Meanwhile, the DMA curves of SA-45G showed minimal change across temperatures, reinforcing the interpretation from DSC analysis (Figure 10f).

Together, these results demonstrate that the thermal response of alginate-based films is highly dependent on plasticizer content, with higher glycerol concentrations conferring greater thermal stability under elevated processing conditions. This underscores the importance of optimizing both plasticizer concentration and thermal profile to preserve film integrity during continuous extrusion.

#### 3.2.2. Mechanical Properties

**Figure 11:**
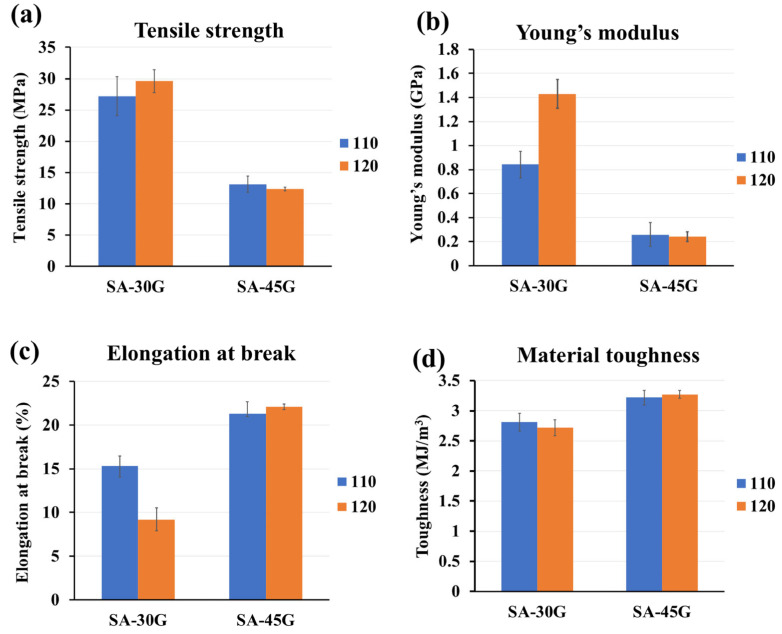
Mechanical properties (**a**) Tensile strength, (**b**) Young modulus, (**c**) Elongation at break, and (**d**) material toughness of the films plasticized with 30 and 45%glycerol processed at two temperature profiles (110 and 120 °C).

Tensile testing was conducted to evaluate how different processing temperature profiles influence the mechanical properties of glycerol-plasticized alginate films (Figure 11). The measured parameters included tensile strength (TS), Young’s modulus (YM), elongation at break (EB), and material toughness (MT) (Figure 11a–d).

For films plasticized with 30% glycerol (SA-30G), an increase in processing temperature led to higher TS and YM, indicating increased stiffness and chain densification, likely due to water loss and tighter molecular packing. However, EB decreased markedly, suggesting reduced flexibility and possible thermal degradation at elevated temperatures. This is consistent with a drop in *T_g_*_2_ observed in the DSC and DMA results, supporting the hypothesis that partial degradation and network rearrangement contributed to embrittlement. Despite the decrease in flexibility, toughness remained relatively unchanged, possibly due to enhanced interactions between the alginate and glycerol at higher temperatures.

In contrast, films containing 45% glycerol (SA-45G) exhibited stable mechanical properties across both processing conditions. A slight increase in EB and toughness at the higher temperature may reflect improved plasticization and enhanced molecular dispersion. The presence of sufficient glycerol likely buffered thermal stress and maintained polymer chain mobility, resulting in a more homogeneous and flexible network.

These trends highlight the importance of glycerol content in moderating the impact of processing temperature. At low glycerol levels, alginate is more susceptible to degradation and embrittlement under thermal stress, whereas higher plasticizer content promotes thermal stability and flexibility.

Similar behavior has been reported for other polysaccharides, such as starch. For instance, Thunwall et al. [69] observed that increasing the processing temperature during compression molding of potato starch improved flexibility (higher EB) but reduced TS, with 140 °C yielding the most plasticized samples. However, starch has a higher degradation temperature and different structural properties compared to alginate. Due to the limited literature on extruded alginate systems, such comparisons help contextualize the temperature sensitivity observed in our study.

Overall, these findings underscore the need for precise control of processing conditions—particularly temperature and water content—in the continuous extrusion of alginate films. Balancing processability and material performance requires optimizing both plasticizer concentration and thermal profile.

## 4. Conclusions

This study introduces a novel and scalable method for producing extrudable alginate-based films via continuous thermo-mechanical extrusion. By systematically varying glycerol concentration and processing temperature, we established key structure–property relationships essential for tailoring alginate materials for bio-based applications. ^1^H NMR confirmed a G-block–rich alginate structure, contributing to its inherent brittleness. Glycerol acted as an effective plasticizer by forming hydrogen bonds with the polymer matrix, as evidenced by FTIR. Increasing glycerol content reduced glass transition, melting, and degradation temperatures, as shown by DSC and DMA. Mechanical testing revealed that higher glycerol levels decreased tensile strength and modulus but significantly increased elongation at break (EB). Processing temperature affected film properties primarily at lower plasticizer levels, where elevated temperatures led to reduced *T_g_* and EB, likely due to partial thermal degradation. In contrast, films with 45% glycerol maintained stable thermal and mechanical properties across both temperature profiles. These findings highlight the importance of optimizing plasticizer content and processing parameters—such as temperature, screw speed, residence time, and feed rate—to achieve desired material performance. This work provides a foundational framework for the scalable production of alginate-based films and contributes to the development of biodegradable alternatives to conventional plastics. Future studies may focus on enhancing film performance further and investigating processing behavior in more detail. The approach presented here holds promise for applications in sustainable packaging, food contact materials, and pharmaceuticals.

## Figures and Tables

**Table 1:** Formulation of the prepared alginate-based materials.

Sample	Alginate/Glycerol (*w*/*w*)	Alginate/Water (*w*/*w*)	Temperature Profile	Mass Fraction of the Components for the Samples Conditioned at the Relative Humidity of 10% and 25 °C		
				x_A_ *	x_G_ *	x_W_ *
**SA**	100/0	65/35	1	0.930	0	0.067
**SA-30G**	70/30	70/30	1&2	0.672	0.282	0.046
**SA-40G**	60/40	75/25	1	0.564	0.380	0.056
**SA-45G**	55/45	80/20	1&2	0.520	0.422	0.058
**SA-50G**	50/50	85/15	1	0.470	0.470	0.060

* x_A_, x_G_, and x_W_ represent the mass fractions of Alginate, Glycerol, and Water in the film, respectively.

**Table 2:** Composition data of studied alginate extracted from ^1^H NMR spectrum.

Composition	Value
**F_G_**	0.53 ± 0.06
**F_M_**	0.47 ± 0.03
**F_GG_**	0.144 ± 0.009
**F_MG_ = F_GM_**	0.386 ± 0.02
**F_MM_**	0.084 ± 0.05
**M/G**	0.89 ± 0.12
**η**	1.55 ± 0.01

**Table 3:** Gordon–Taylor constants (*K_AG_*, *K_AW_*, and *T_gA_*) obtained by fitting data obtained from DMA measurements to evaluate the plasticizing effect of glycerol on the glass transition of alginate.

Gordon-Taylor Parameters	*K_AG_*	*K_AW_*	*T_gA_* (°C)	*E*
**Value**	0.9	2.3	156	3.85

## Data Availability

Data are contained within the article.
